# Trends in obesity-related cardiovascular and cancer mortality in Switzerland 1995-2019: an analysis of multiple causes of death

**DOI:** 10.1093/aje/kwag003

**Published:** 2026-01-08

**Authors:** Bernadette W A van der Linden, Célia A Viehl, Nazihah Noor, Tim Adair, Salvatore Vaccarella, Cristian Carmeli

**Affiliations:** Population Health Laboratory (#PopHealthLab), University of Fribourg, Fribourg, Switzerland; Population Health Laboratory (#PopHealthLab), University of Fribourg, Fribourg, Switzerland; Population Health Laboratory (#PopHealthLab), University of Fribourg, Fribourg, Switzerland; Nossal Institute for Global Health, Melbourne School for Population and Global Health, University of Melbourne, Carlton, Australia; Cancer Surveillance Branch, International Agency for Research on Cancer (IARC/WHO), Lyon, France; Population Health Laboratory (#PopHealthLab), University of Fribourg, Fribourg, Switzerland

**Keywords:** cancer epidemiology, cardiovascular disease (CVD), mortality rate, obesity, public health surveillance, temporal trend

## Abstract

Obesity increases cardiovascular disease (CVD) and cancer mortality risk, with prevalence rising globally over recent decades. In the United States, steep obesity increases contributed to adverse trends in obesity-related mortality and to slowing decline in overall CVD mortality, particularly among younger generations. Switzerland experienced slower obesity increases, but the contribution of obesity to mortality trends remains uncharacterized. We analyzed all adult deaths recorded in Swiss mortality statistics between 1995 and 2019. Obesity-related CVD and cancer deaths were identified using multiple cause of death approaches. Annual changes in age-standardized mortality rates were estimated via segmented regression. Age-period-cohort models assessed cohort variations. Overall, CVD mortality declined steadily while cancer mortality decline attenuated after 2005, primarily reflecting slower declines in obesity-unrelated cancer mortality. Obesity-related mortality increased from 1995 to 2005 and then decreased, while obesity-unrelated rates decreased throughout 1995-2019. These diverging trends did not slow overall CVD mortality decline. Age-period-cohort modeling revealed lower obesity-related mortality rates in younger versus older generations. In Switzerland, unlike in the United States, trends in obesity-related mortality did not slow the decline of overall CVD mortality. Obesity-related mortality rates did not increase in younger generations, highlighting the role of reduced childhood obesity prevalence and improved management of obesity-related conditions in Switzerland.

## Introduction

The increasing prevalence of obesity represents a significant public health challenge,^1^ as obesity constitutes the third-largest contributor to disease burden in Western Europe^2^ and substantially increases risk of adult mortality from cardiovascular diseases (CVDs) and cancers—the leading causes of death worldwide.^3^ Obesity is especially concerning in countries with high prevalence, such as the United States and Australia, where it has been a major contributor to the reversal of long-term decline in CVD mortality rates since 2010.^4^^,^^5^ In these countries, obesity-related CVD deaths have increased accounting for up to 50% of overall CVD mortality in the United States.^4^ The largest increases occurred among younger decedents aged 35-44, indicating heightened obesity-related CVD mortality risk among recent birth cohorts.^4^ Conversely, among individuals whose deaths were unrelated to obesity, CVD mortality rates continued to decline steadily until 2017 in both countries. For cancer mortality in the United States, the long-term decline in overall rates accelerated after 2011, primarily due to similar trends in obesity-unrelated mortality.^6^ However, obesity-related cancer mortality rates, which account for one-third of overall cancer deaths, slowed their decline since 2011, particularly among women.^6^ Additionally, the incidence of some obesity-related cancers between 1995 and 2014 has risen in successively younger generations.^7^

Switzerland has also experienced rising obesity (body mass index ≥30) prevalence in recent decades, though at a slower pace than the United States.^8^ Among adult men, the obesity prevalence in the United States rose from 18% in 1980 to 26.6% in 2000 and 31.7% in 2013, while in Switzerland it increased from 15.2% in 1980 to 16.3% in 2000 and 18.4% in 2013. Similar patterns were observed among women.^8^ Notably, while childhood obesity trends remained stable at approximately 7% in Switzerland over this period, they increased from 7% in 1980 to about 13% in 2013 in the United States. During this same period, for both men and women death rates from coronary heart disease and stroke decreased in Switzerland,^9^ and overall cancer mortality rates declined.^10^^-^^12^ However, no assessment has examined obesity’s contribution to CVD and cancer mortality trends in Switzerland, particularly whether obesity-related and obesity-unrelated mortality rates diverged and whether these trends differed across generations.

To address this knowledge gap, we examined trends in obesity-related and obesity-unrelated CVD and cancer mortality rates in Switzerland from 1995 to 2019. We utilized multiple cause of death (MCOD) data as they provide an effective approach for identifying obesity-related and obesity-unrelated causes of death.^4^^,^^13^^,^^14^

## Methods

### Study population and data source

The target population comprised individuals aged 20 and older residing in Switzerland. The study population included all deaths that occurred between 1995 and 2019 and were recorded in the mortality database of the Swiss Federal Statistical Office.^15^ We excluded years after 2019 as the COVID-19 pandemic introduced substantial short-term mortality disruptions that may complicate interpretation of long-term obesity-related mortality trends. We extracted individual-level data on specific causes of death, year and age at death, sex, and annual population size. Individual cause of death data encompassed both underlying and immediate causes of death (recorded in Part I of the certificate) and up to two secondary causes related to contributing diseases (recorded in Part II of the certificate).

### Obesity-related CVD and cancer mortality

We identified CVD and cancer deaths using *ICD-10* codes specified in the CVD and neoplasm chapters of the Global Burden of Disease (GBD) Study 2019 (detailed in Appendix S1).^16^

Obesity-related deaths were identified using an MCOD approach based on mentions of diabetes, chronic kidney disease, obesity, dyslipidemias, or hypertensive heart disease (henceforth labeled DKOLH) among the reported causes of death listed on death certificates.^4^ Beyond obesity, the remaining DKOLH conditions were selected based on four main reasons. First, a substantial body of observational and experimental evidence demonstrates the increased risk for these conditions conferred by high body mass index or excess weight.^17^^-^^23^ Second, the GBD study estimates that, among individuals aged 20 + years in Switzerland, 45% of deaths with hypertensive heart disease as the underlying cause, 42% with diabetes, and 24% with chronic kidney disease are attributable to high body mass index.^24^ These are the causes of death with the three highest percentages of deaths attributable to high body mass index, excluding causes with much lower total deaths.^24^ Third, during 1995-2019 in Switzerland, 46% of deaths with obesity (*n* = 18 198) had at least one of these conditions reported in the death certificate, compared to 25% for deaths without a mention of obesity (*n* = 1 710 324). This demonstrates that these conditions frequently co-occur with obesity, supporting their use as obesity-related causes of death*.* Fourth, these conditions were used in previous research^4^^,^^14^^,^^25^^,^^26^ (*ICD-10* codes are reported in Appendix S2), thereby enabling comparison with trends estimated in the United States and Australia.

We first identified all deaths in which CVD or cancer were listed as causes recorded on death certificates. These deaths were then categorized into two groups: (1) DKOLH CVD/cancer deaths, where at least one predefined DKOLH condition was also listed on the death certificate, and (2) non-DKOLH CVD/cancer deaths, where none of the DKOLH conditions were mentioned on the death certificate.^4^ Notably, we did not examine immediate causes of death and focused on underlying and contributing causes to emphasize distinct causal pathways originating from the primary morbid process initiated by the underlying cause.^27^

### Statistical analyses

We estimated annual CVD and cancer mortality rates using a weighted MCOD approach. While the traditional approach assigns a weight of one to the underlying cause of death (UCOD) and zero to all other causes present in the certificate, the weighted MCOD approach distributes fractional weights across all causes and maintains the individual death as the unit of analysis with weights summing to one. In our weighting scheme, deaths listing CVD or cancer as the underlying cause received twice the weight of those listing these conditions as contributing causes. For instance, in a death certificate documenting three causes, CVD or cancer received a weight of 0.5 when listed as the underlying cause, but only 0.25 when listed as a contributing cause. By prioritizing the UCOD, this approach aligns with established public health conventions while also incorporating contributing causes. Thus, our weighted MCOD approach represents a balanced compromise between the traditional hierarchy of causes and the complexity of multimorbidity. We implemented this “double UCOD” weighting strategy, following algorithms described in previous MCOD research.^28^^,^^29^

We calculated mortality rates stratified by sex, for the overall population as well as for specific age groups: 20-44 years, 45-59 years, 60-74 years, and 75 years and older.^9^ We age-standardized the mortality rates by applying the direct standardization method using the 2013 European standard population.

To estimate the annual percentage change in mortality rates, we ran separate segmented regressions (JoinPoint Regression Program)^30^ for CVD and cancer mortality, each stratified by sex and DKOLH vs. non-DKOLH conditions (8 regressions total). The algorithm allowed up to two join points, dividing the 1995-2019 period into a maximum of three segments. Within each segment, a linear model was fitted to the log-transformed mortality rates, where the slope corresponds to the annual percentage change in mortality rates. The optimal number of join points was selected based on Monte Carlo permutation tests (5% significance level). Uncertainty in annual percentage changes was calculated via bootstrap resampling (1000 replications) and reported with 95% compatibility intervals (CI).

Period- and cohort-based variations in the mortality rates trends were assessed by fitting age-period-cohort models.^31^ If the contribution of obesity to mortality was greater for younger birth cohorts, we expect to observe cohort-based increases in DKOLH death rates among more recent birth cohorts. In contrast, if obesity contributed to mortality across all age groups, we expect to observe period-based increases of DKOLH death rates. We fitted all models using 10 age groups (20-44, 45-49, 50-54, 55-59, 60-64, 65-69, 70-74, 75-79, 80-84, 85+) and 5 period groups (1995-1999, 2000-2004, 2005-2009, 2010-2014, 2015-2019), resulting in 50 age × period cells covering 14 birth cohorts groups (period—age = cohort). We implemented the intrinsic estimator as it is an appropriate and widely used constraint for age-period-cohort models^32^^-^^34^ (Appendix S3).

### Sensitivity analyses

First, to evaluate the impact of our weighting strategy, we estimated mortality rates by assigning equal weights to each cause of death reported on the certificate.^27^ Second, to address potential misclassification of CVD deaths due to *ICD-10* garbage codes, we redistributed relevant garbage codes listed as the UCOD to CVD deaths and recalculated age-standardized mortality rates^35^ (Appendix S4). We prioritized CVD over cancer for this analysis because the proportion of garbage codes potentially related to CVD substantially exceeds that for cancer.^36^^,^^37^ Third, to assess the validity of the chosen age-period-cohort models to statistically identify period/cohort-based variations in mortality trends, we applied the intrinsic estimator by changing the model specification^33^ (Appendix S5). Finally, to validate our definition of obesity-related cancers against established criteria, we assessed the concordance between our approach and that implemented in previous research^6^ following the International Agency for Research on Cancer (IARC) framework^38^ (Appendix S6).

## Results

### Trends in overall CVD and cancer mortality

Between 1995 and 2019, there were 1 581 135 deaths from all causes (52% women), of which 640 617 (40.5%) were related to CVD and 459 222 (29%) to cancer, either as the underlying cause or as a contributing cause of death.

**Figure 1:**
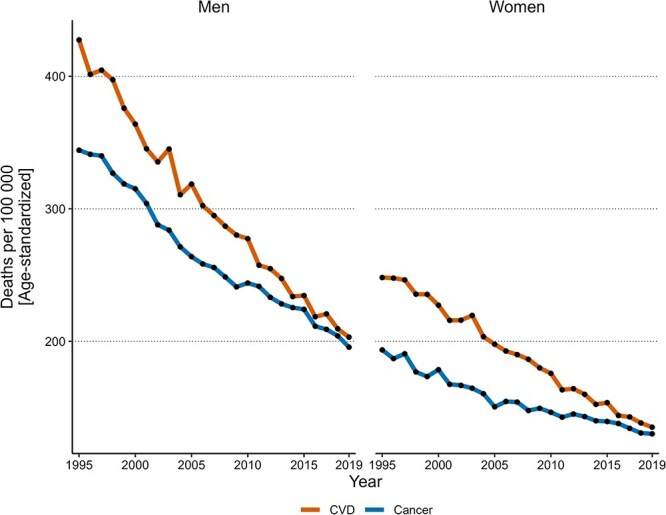
Age-standardized CVD and cancer mortality rates 1995-2019 by sex. Black dots are the measured year; color lines are from linear interpolation between dots. Abbreviation: CVD, cardiovascular disease.

The age-standardized mortality rates from CVD and cancer declined throughout 1995-2019 (Figure 1 displays MCOD-based rates, while Figure S1 UCOD-based rates). The decline was faster for CVD than for cancer and was steady for CVD while it has been attenuated since 2005 for cancer. Among men, the CVD mortality rate decreased from 427 to 203 per 100 000 between 1995 and 2019 (Table 1), corresponding to a steady decline (ie, a negative annual percentage change as estimated by the JoinPoint regression) of 3% (95% CI, 2.9%-3.1%). For cancer, the mortality rate decreased from 344 to 196 per 100 000 over the same period. This corresponded to an annual decline of 2.8% (95% CI, 2.4%-3.4%) between 1995 and 2005 (join point estimated by the JoinPoint regression), which then slowed to 2.0% (95% CI, 1.4%-2.2%) from 2005 to 2019. Among women, the CVD mortality rate declined from 248 to 135 per 100 000 between 1995 and 2019, corresponding to a steady annual decline of 2.6% (95% CI, 2.5%-2.8%). For cancer, the mortality rate declined from 193.5 to 130 per 100 000 over the same period. This corresponded to an annual decline of 2.1% (95% CI, 1.7%-2.8%) from 1995 to 2005, attenuating to 1.2% (95% CI, 0.7%-1.4%) between 2005 and 2019.

**Table 1:** Age-standardized mortality rates over time and by sex.

	**Men**			**Women**		
	**1995**	**2005**	**2019**	**1995**	**2005**	**2019**
CVD mortality	427	319	203	248	198	135
DKOLH	59 (14%)	95 (30%)	69 (34%)	47 (19%)	74 (37%)	54 (38%)
Non-DKOLH	368 (86%)	224 (70%)	134 (66%)	201 (81%)	124 (63%)	81 (62%)
Cancer mortality	344	264	196	193.5	150.5	130
DKOLH	17.5 (5%)	41 (15.5%)	34 (17%)	11 (6%)	22 (15%)	19.5 (15%)
Non-DKOLH	326.5 (95%)	223 (84.5%)	162 (83%)	182.5 (94%)	128.5 (85%)	110.5 (85%)

Abbreviations: CVD, cardiovascular disease; DKOLH, diabetes, chronic kidney disease, obesity, dyslipidemias, hypertensive heart disease. Age-standardized rates are reported for 100 000 persons. Besides the first and last year of the examined period, 2005 is the join point estimated by the JoinPoint regression for the trends in both overall CVD and cancer mortality rates. Proportions (%) of DKOLH and non-DKOLH mortality are reported in parenthesis.

### Trends in DKOLH and non-DKOLH cardiovascular and cancer mortality

Diabetes, chronic kidney disease, obesity, dyslipidemias, and/or hypertensive heart disease CVD deaths accounted for 223 948 deaths, representing 35% of all identified CVD deaths, while DKOLH cancer deaths totaled 83 021, making up 18% of all identified cancer deaths. Diabetes and hypertensive heart disease appeared in more than 30% and 60% of DKOLH CVD or cancer deaths, respectively. Chronic kidney disease, obesity, and dyslipidemias each appeared in less than 10%. Finally, CVD or cancer deaths with at least one mention of either diabetes or hypertensive heart disease accounted for 90% of DKOLH deaths.

**Figure 2:**
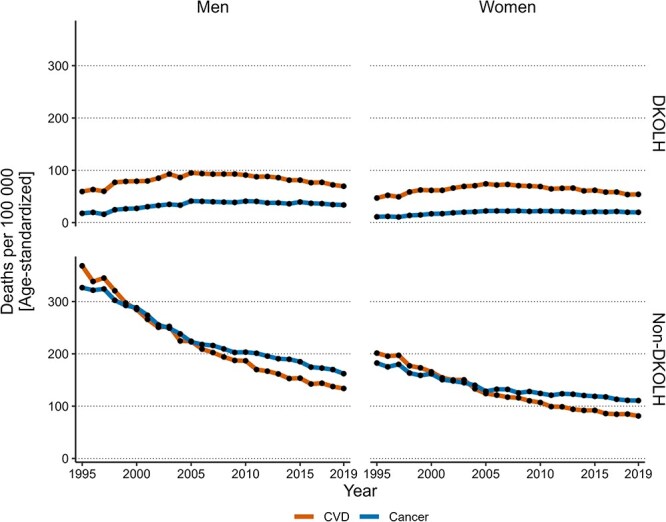
DKOLH and non-DKOLH mortality trends by sex. Age-standardized DKOLH (top) and non-DKOLH (bottom) CVD and cancer mortality rates from 1995-2019 by sex (men on the left, women on the right). Abbreviations: CVD, cardiovascular disease; DKOLH, diabetes, chronic kidney disease, obesity, dyslipidemias, hypertensive heart disease.

DKOLH CVD and cancer rates increased from 1995 to 2005, followed by a decline thereafter (Figure 2). The decline was faster for CVD compared to cancer. Between 2005 and 2019, among men CVD rates decreased yearly by 2.1% (95% CI, 1.5%-2.8%) while cancer rates decreased by 1.1% (95% CI, 0.05%-2.4%). Similarly, among women, CVD rates decreased yearly by 2.3% (95% CI, 1.8%-2.8%) while cancer rates declined by 1.0% (95% CI, 0.3%-1.7%). Analysis of mortality rates related to each individual DKOLH condition (Figure S2) revealed similar patterns of initial increases followed by declines for deaths involving diabetes or hypertensive heart disease. Mortality rates for these conditions were the highest in magnitude.

In contrast, age-standardized non-DKOLH mortality rates for CVD and cancer declined during the whole period, with a sizeable attenuation of the pace of decline occurring between 2005 and 2011 (as estimated by the JoinPoint regression). Among men, CVD mortality rates decreased yearly by 5.0% (95% CI, 4.5%-6.0%) from 1995 to 2006, compared to 3.5% (95% CI, 2.7%-3.8%) from 2006 to 2019. Cancer mortality rates declined yearly by 3.8% (95% CI, 3.4%-4.6%) from 1995 to 2006, compared to 2.2% (95% CI, 1.6%-2.5%) from 2006 to 2019. Among women, CVD mortality rates declined yearly by 4.4% (95% CI, 4.2%-5%) from 1995 to 2011, compared to 2.5% (95% CI, 1.5%-3.3%) from 2011 to 2019. Cancer mortality rates declined yearly by 3.2% (95% CI, 2.7%-3.9%) from 1995 to 2005, compared to 1.3% (95% CI, 0.9%-1.6%) from 2005 to 2019.

Site-specific analysis of non-DKOLH cancer mortality (Figure S3) revealed variations in these trends: lung cancer deaths increased consistently among women, while the pace of decline attenuated for colorectal cancer deaths in both sexes, breast cancer deaths among women, and pancreatic cancer deaths among men.

Overall, because of these differing trends between DKOLH and non-DKOLH mortality rates, the proportion of DKOLH CVD or cancer deaths within overall CVD or cancer mortality increased between 1995 and 2019 for both men and women (Table 1). Diabetes, chronic kidney disease, obesity, dyslipidemias, and/or hypertensive heart disease CVD rates among men were 14% of the total CVD mortality rate in 1995 and rose to 34% in 2019. Among women, these proportions rose from 19% in 1995 to 40% in 2019. Notably, these proportions were higher among women than men. Diabetes, chronic kidney disease, obesity, dyslipidemias, and/or hypertensive heart disease cancer rates among men were 5% of the total cancer mortality rate in 1995, while they rose to 17% in 2019. Among women, these proportions rose from 6% in 1995 to 15% in 2019.

Throughout the study period, DKOLH CVD rates were consistently higher than DKOLH cancer rates. In contrast, non-DKOLH CVD mortality rates were higher than cancer mortality rates in 1995 then fell lower by 2019 due to the higher pace of decline in CVD mortality compared to cancer mortality.

Trends in DKOLH mortality rates observed for all adult ages combined were most pronounced in the 75+ age group for CVD and in the 60+ groups for cancer (Figure S4). In contrast, CVD and cancer rates in younger age groups remained relatively stable or slowly decreasing. Together, these age-specific trends point to both period-based and cohort-based variations in DKOLH CVD and cancer mortality trends. Non-DKOLH mortality rates decreased steadily across most age groups (Figure S5), except for CVD rates in the 60-74 age group and cancer rates in the 75+ age group, where the decline markedly attenuated in the 2010s.

### Period and cohort variations in mortality rates

**Figure 3:**
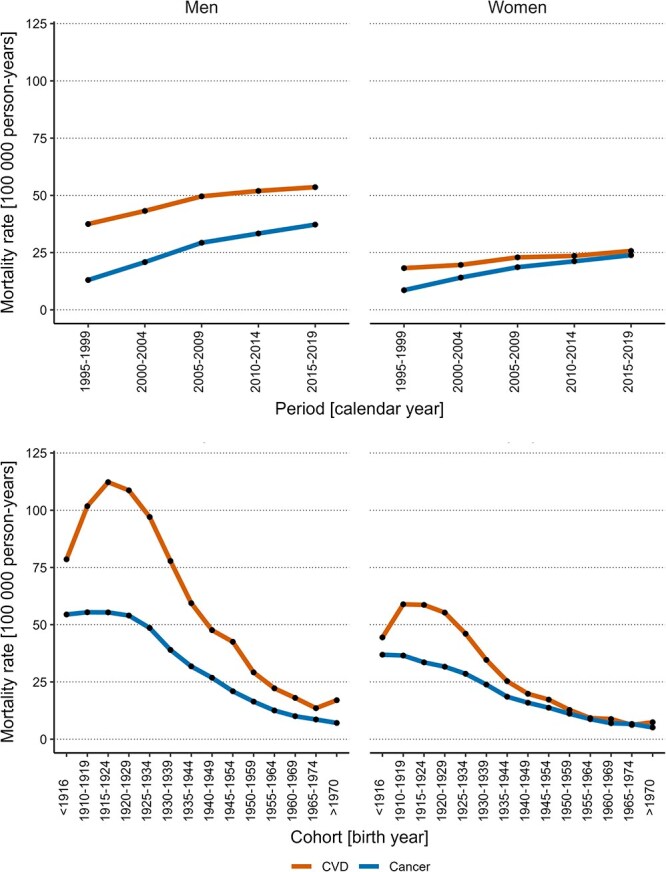
Period- and cohort-based variations in DKOLH mortality rates by sex. Period-based (top) and cohort-based (bottom) variations in DKOLH CVD and cancer mortality rates by sex (men on the left, women on the right). Abbreviations: CVD, cardiovascular disease; DKOLH, diabetes, chronic kidney disease, obesity, dyslipidemias, hypertensive heart disease.

**Figure 4:**
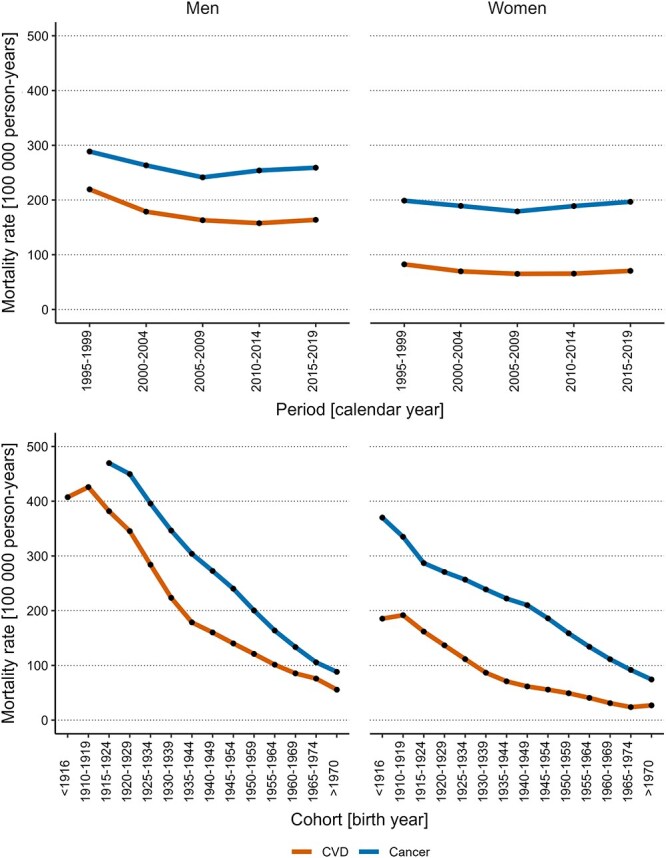
Period-based and cohort-based variations in non-DKOLH mortality rates by sex. Period-based (top) and cohort-based (bottom) variations in non-DKOLH CVD and cancer mortality rates by sex (men on the left, women on the right). Abbreviations: CVD, cardiovascular disease; DKOLH, diabetes, chronic kidney disease, obesity, dyslipidemias, hypertensive heart disease.

There were substantial variations in both period-based and cohort-based rates of DKOLH deaths (Figure 3). Specifically, period-based mortality rates increased steadily between 1995 and 2019. Cohort-based mortality rates increased (for CVD) or remained stable (for cancer) among decedents born before 1929, while decreasing steadily among those born after 1929. In contrast, for non-DKOLH deaths, there were substantial variations in cohort-based mortality rates only (Figure 4), whereby cohort-based rates decreased substantially. While variations in period-based rates were smaller, there was a trend of decreasing period-based rates until 2005-2009 followed by an increasing trend for non-DKOLH cancer mortality. Age-based CVD and cancer mortality rates increased exponentially with older age (Figure S6).

### Sensitivity analyses

When using equal weights across causes of deaths or redistributing relevant garbage codes to CVD, trends in DKOLH and non-DKOLH CVD/cancer mortality remained similar to the main analysis (Figures S7 and S8).

When applying the intrinsic estimator with different age-period-cohort specifications, estimated period/cohort-based variations were similar to main results (Figures S9 and S10).

IARC-defined obesity-related cancers comprised 40.8% of MCOD-based obesity-related deaths versus 38.1% of obesity-unrelated deaths (Fisher’s test odds ratio = 1.12, *P* < 2^-16^), supporting concordance between the two operationalizations.

## Discussion

This study examined trends of obesity-related and -unrelated CVD and cancer mortality rates in Switzerland from 1995 to 2019, using MCOD data. Our analysis yielded three key findings. First, while both CVD and cancer mortality rates declined over the study period, the patterns differed markedly. CVD mortality showed a consistent downward trend from 1995 to 2019, whereas cancer mortality decline attenuated after 2005. This deceleration in cancer mortality reduction was primarily associated with a slowdown in the decline of non-DKOLH cancer rates. Second, DKOLH mortality exhibited less favorable trends compared to non-DKOLH mortality. Diabetes, chronic kidney disease, obesity, dyslipidemias, and/or hypertensive heart disease mortality rates increased between 1995 and 2005 before declining thereafter, while non-DKOLH mortality declined throughout 1995-2019. Consequently, the relative share of DKOLH deaths within overall mortality increased substantially, particularly for CVD. The proportion of DKOLH CVD deaths rose by 20 percentage points—from 14% to 34% among men and from 19% to 40% among women between 1995 and 2019. For cancer, the corresponding increase was 10 percentage points. Third, DKOLH CVD and cancer mortality rates decreased substantially across successive birth cohorts.

The steady decline in overall CVD mortality observed in this study did not attenuate, as the adverse trend observed for DKOLH CVD mortality was offset by a strong decline in non-DKOLH CVD mortality rates. These findings contrast with those from other high-income countries.^5^ In particular, in the United States and Australia trends in DKOLH CVD mortality were too adverse to be offset by the decline in non-DKOLH rates, leading to an attenuation of the long-term decline in overall CVD mortality since 2011 and 2005, respectively.^4^ These differences in DKOLH rates may be explained by country-wide variations either in obesity prevalence and incidence or in medical treatment and control of obesity-related conditions. On one hand, the obesity prevalence in the United States and Australia has increased by more than 10 percentage points, from less than 20% in 1980 to over 30% in 2013,^8^ while in Switzerland it increased by 5% from about 15% in 1980 over the same period. On the other hand, more effective population-wide treatment and management of DKOLH conditions in Switzerland could also have contributed to a smaller impact of obesity on CVD mortality. Notably, hypertension treatment and control improved in Switzerland during the 2000s.^39^^,^^40^ This is particularly relevant, as hypertensive heart disease deaths accounted for the largest proportion of DKOLH deaths and exhibited corresponding declines during this period. Compared to the United States, control of type 2 diabetes and hypertension was achieved in a similar portion of the population in 2007, while control of hypercholesterolemia was higher—75% in Switzerland vs 52% in the United States.^39^^,^^41^ Compared to Australia, control of hypertension was achieved in a larger share of the population in 2007—80% in Switzerland vs 32% in Australia.^42^

In contrast to the trends of CVD mortality rates, the decline in cancer mortality slowed from 2005 onward. This attenuation is unlikely to be attributed only to the adverse trend in DKOLH cancer mortality rates, as (1) the DKOLH rates accounted for up to approximately 15% of the overall cancer rates; (2) between 2005 and 2019 the pace of decline for DKOLH rates was similar to that of non-DKOLH rates; and (3) non-DKOLH cancer mortality rates also attenuated starting in 2005 for women and in 2006 for men. Thus, the observed attenuation in overall cancer mortality decline is mainly attributable to a slowdown in the decline of non-DKOLH cancer mortality. Our analysis of non-DKOLH cancer mortality by specific cancer sites revealed a slowdown in declining mortality for various cancer sites in both sexes, and an increase in lung cancer mortality among women. Additionally, the age-period-cohort modeling indicated this slowdown of non-DKOLH trends to be period-based rather than cohort-based. As such, the role of increasing socioeconomic inequalities in cancer mortality and increasing lung cancer mortality among women warrant closer examination. Recent research reported an increase in educational inequalities in cancer mortality across various European countries, including Switzerland, during 1990-2015.^43^ Increases in tobacco smoking prevalence among women in Switzerland have led to a rise in lung cancer incidence and mortality.^44^ This is in contrast with the United States, where smoking prevalence has been declining steadily since the 1970s among adults.^45^ Notably, this smoking-related difference could partly explain the slowdown in non-DKOLH cancer rates in Switzerland, while rates in the United States continued to decline more rapidly.^46^

The trends for DKOLH mortality were worse than those for non-DKOLH mortality, as DKOLH mortality increased between 1995 and 2005 and declined afterwards, while non-DKOLH mortality declined throughout the examined period. Notably, DKOLH CVD mortality rates were consistently higher than DKOLH cancer mortality rates. Additionally, while the proportion of DKOLH deaths within overall mortality increased during the examined period, it increased more for CVD than for cancer. These findings may signal a changing profile of overall CVD mortality as the consequences of the obesity epidemic emerge. However, this remains uncertain and will depend on the dynamic of the obesity epidemic and on the effectiveness of the pharmaceutical control of obesity-related conditions.

Finally, DKOLH CVD and cancer mortality trends were decomposed into a period-based upward variation and a cohort-based complex variation, which is upward for those born before 1929 and downward for later birth cohorts. The period-based variation aligns with an increase in prevalence of diabetes, hypertension, and hypercholesterolemia between 1997 and 2007 among Swiss adults^40^ and findings from studies in the United States showing a rise in obesity prevalence across all adult age groups.^31^^,^^47^^,^^48^ The observed cohort-based variations mirror an increase in obesity prevalence between 1982 and 2007 in Switzerland, particularly among adults born before 1939.^46^ However, they are in contrast with the rise in obesity self-reported by Swiss residents born between 1960 and 1979,^46^ and the increased obesity-related cancer incidence and CVD mortality among recent birth cohorts in the United States.^4^^,^^7^ Our finding of substantially lower DKOLH mortality rates across successive birth cohorts may be explained by two factors. First, treatment and control of hypertension and hypercholesterolemia greatly improved in Switzerland during the 2000s,^39^^,^^40^ potentially benefiting those middle aged and born in the 1960s and onward and thus mitigating their obesity-related mortality risk. Second, contrary to the United States, where childhood obesity rose since the late 1990s, Switzerland experienced an epidemic of adult obesity while childhood obesity remained stable around 5-6%.^8^ Thus, the population was not exposed to obesity for a longer duration across the life course, which may have contributed to keeping obesity-related mortality low among younger generations.

Strengths of this study are (1) complete coverage of death data in Switzerland,^15^ thereby our findings are generalizable to the target Swiss adult population, and (2) the use of the MCOD approach to identify obesity-related deaths. This approach estimated a higher proportion of DKOLH CVD mortality rates compared to cancer mortality, in line with previous studies implementing a different statistical method to estimate the contribution of obesity to mortality, and with extensive evidence of obesity’s role in causing CVD mortality.^18^^,^^20^ Additionally, this proportion was higher among women than men, highlighting the established increased susceptibility of obese women to CVDs.^49^ MCOD data provide a more direct, individual-level proxy by using information recorded on death certificates compared to estimated mortality fractions from survey data.^4^^,^^14^ The latter approach uses national survey data on obesity prevalence and all-cause mortality to estimate obesity-attributable mortality fractions but is prone to confounding bias when estimating the long-term effects of obesity on mortality.^20^ Additionally, the MCOD approach is valuable in capturing the many co-morbid conditions that contribute to adult CVD and cancer mortality, and relying solely on a single underlying cause may not fully capture the complexity of mortality, as multiple contributing factors often play a role.^50^^-^^54^

This study has limitations. First, our definition of obesity-related causes of death may not capture only mortality caused by obesity, because the examined DKOLH conditions could also be caused by other risk factors; or may not capture some deaths caused by obesity in case the examined conditions were absent from the death certificate. Relatedly, long-term changes in obesity may be accompanied by improvements in early detection and treatment of disease; for cancer, this scenario could increase cancer incidence while decreasing mortality for some cancer types. We attempted to address these challenges by disentangling period-based and cohort-based variations in mortality rates, and by triangulating evidence across two different groups of diseases.^55^ Longitudinal data may help to more precisely define how obesity may have been associated with trends in both DKOLH CVD and cancer mortality, including the impact of improved treatment and control of hypertension and hypercholesterolemia during the 2000s. Second, there could be some missing or misclassified conditions among the causes reported in the death certificate. A study comparing causes of deaths coding with hospital in-patient records throughout 2010-2012 among Swiss residents, reported that for 83% of decedents the UCOD could be identified among hospital diagnoses, while for 77% of decedents the main hospital diagnosis was reported among the causes of death recorded on the death certificate.^56^ Third, the Swiss death certificate is peculiar as it includes only up to two contributing diseases while international death certificates report more. This limited recording may lead to underreporting of comorbidities, thereby underestimating their contribution in MCOD analyses. However, previous studies suggest that physicians tend to prioritize the most clinically significant conditions when constrained by space,^57^ thus our analysis captures the most relevant diseases contributing to death.

In conclusion, this study demonstrates that CVD and cancer mortality rates declined in Switzerland throughout 1995-2019, though with distinct patterns. While CVD mortality improved consistently, cancer mortality decline decelerated after 2005, primarily reflecting a slowdown in obesity-unrelated cancer mortality reduction. Notably, obesity-related CVD and cancer mortality exhibited less favorable trends than obesity-unrelated mortality, increasing the proportion of obesity-related deaths. Given the ongoing obesity pandemic, these findings underscore the importance of continued surveillance of CVD and cancer mortality in Switzerland. Our results also reveal an encouraging contrast with the United States and Australia: in Switzerland, obesity-related mortality rates have not increased among younger generations, likely reflecting lower childhood obesity prevalence and more effective management of obesity-related conditions.

This study also highlights the value of MCOD data for tracking complex mortality trends, providing insights across diseases and demographic groups for public health planning and intervention strategies.

## Supplementary Material

Web_Material_kwag003

## Data Availability

The data underlying this article were provided by the Swiss Federal Statistical Office under license/by permission. Data will be shared on request to the corresponding author with the permission of the Swiss Federal Statistical Office.
