# Supplementary material for: Trends in obesity-related cardiovascular and cancer mortality in Switzerland 1995-2019: an analysis of multiple causes of death
Source: Am J Epidemiol. 2026 Jan 8;195(5):1319–28. doi: 10.1093/aje/kwag003 (PMC13149030; doi:10.1093/aje/kwag003)
Supplement: Web_Material_kwag003 [file web_material_kwag003.pdf]

Supplementary Data

Trends in obesity-related cardiovascular and cancer mortality in Switzerland 1995–2019: an analysis of multiple causes of death

Bernadette WA van der Linden[1], Célia A. Viehl[1], Nazihah Noor[1], Tim Adair[2], Salvatore Vaccarella[3], Cristian Carmeli[1]

[1] Population Health Laboratory (#PopHealthLab), University of Fribourg, Fribourg, Switzerland
[2] Nossal Institute for Global Health, Melbourne School for Population and Global Health, University of Melbourne, Australia
[3] Cancer Surveillance Branch, International Agency for Research on Cancer (IARC/WHO), Lyon, France

Table of Contents

Appendix S1: ICD-10 codes

Appendix S2: ICD-10 codes of DKOLH conditions

Appendix S3: Age-period-cohort modeling

Appendix S4: Sensitivity analysis with re-allocation of garbage codes

Appendix S5: Sensitivity analysis with a different specification of the intrinsic estimator for the age-period-cohort model

Appendix S6: Sensitivity analysis with a different definition of obesity-related cancers

Figure S1: CVD/cancer mortality rates by sex and by weighting method of death causes

Figure S2: CVD/cancer mortality rates by individual DKOLH condition

Figure S3: Non-DKOLH cancer mortality rates by specific cancer sites

Figure S4: DKOLH CVD/cancer mortality rates by sex and age group

Figure S5: Non-DKOLH CVD/cancer mortality rates by sex and age group

Figure S6: Age-based variations in CVD and cancer mortality rates by sex and obesity

Figure S7: DKOLH and non-DKOLH CVD and cancer mortality rates from 1995-2019 by sex estimated with equal weights across multiple causes of death.

Figure S8: DKOLH and non-DKOLH CVD mortality rates without reassigned garbage codes and with reassigned garbage codes from 1995-2019 by sex

Figure S9: Period-based and cohort-based variations in DKOLH CVD and cancer mortality rates by sex

Figure S10: Period-based and cohort-based variations in non-DKOLH CVD and cancer mortality rates by sex

References

Appendix S1: ICD-10 codes

This study used the Global Burden of Disease Study 2019 (GBD 2019) Cause List. To create the aggregated CVD and cancer list, the following groups from the non-communicable diseases chapter were used:

- Cardiovascular diseases
- Neoplasms

Additionally, garbage codes classified as class 3 and 4 were redistributed to CVD or cancer deaths when possible.

ICD-10 codes	
Cardiovascular diseases	GBD 4-digit codes:<br>B332, G450, G451, G452, G453, G454, G458, G459, G460, G461, G462, G463, G464, G465, G466, G467, G468,<br>I010, I011, I012, I018, I019, I020, I050, I051, I052, I058, I059, I060, I061, I062, I068, I069, I070, I071, I072, I078, I079, I080, I081, I082, I083, I088, I089, I090, I091, I982, I098, I099, I110, I119, I200, I201, I208, I209, I210, I211, I212, I213, I214, I219, I220, I221, I228, I229, I230, I231, I232, I233, I234, I235, I236, I238, I240, I241, I248, I249, I250, I251, I252, I253, I254, I255, I256, I258, I259, I270, I272, I280, I281, I288, I289, I300, I301, I308, I309, I310, I311, I318, I319, I320, I321, I328, I330, I339, I340, I341, I342, I348, I349, I350, I351, I352, I358, I359, I360, I361, I362, I368, I369, I370, I371, I372, I378, I390, I391, I392, I393, I394, I398, I400, I401, I408, I409, I410, I411, I412, I418, I421, I422, I423, I424, I425, I426, I427,<br>I428, I430, I431, I432, I438, I470, I471, I472, I479, I480, I481, I482, I483, I484, I489, I510, I511, I512, I513, I514, I600, I601, I602, I603, I604, I605, I606, I607, I608, I609, I610, I611, I612, I613, I614, I615, I616, I618, I619, I620, I621, I629, I630, I631, I632, I633, I634, I635, I636, I638, I639, I650, I651, I652, I653, I658, I659, I660, I661, I662, I663, I664, I668, I669, I670, I671, I672, I673, I675, I676, I680, I681, I682, I690, I691, I692, I693, I702, I708, I710, I711, I712, I713, I714, I715, I716, I718, I719, I720, I721, I722, I723, I724, I725, I726, I728, I729, I730, I731, I738, I739, I770, I771, I772, I773, I774, I775, I776, I778, I779, I780, I781, I788, I789, I790, I791, I792, I798, I800, I801, I802, I803, I808, I809, I820, I821, I822, I823, I828, I829, I830, I831, I832, I839, I860, I861, I862, I863, I864, I868, I870, I871, I872, I878, I879, I880, I881, I888, I889, I890, I899,<br>K751<br>GBD 3-digit codes:<br>G45, G46, I01, I02, I05, I06, I07, I08, I09, I11, I20, I21, I22, I23, I24, I25, I28, I30, I31, I32, I33, I34, I35, I36, I37, I38, I39, I40, I41, I43, I47, I48, I60, I61, I62, I63, I65, I66, I71, I72, I73, I77, I78, I79, I80, I81, I82, I83, I86, I87, I88, I89, I98<br>Garbage codes redistributed:<br>I379, I42, I420, I429, I44, I440, I441, I442, I443, I444, I445, I446, I447, I45, I450, I451, I452, I453, I454, I455, I456, I458, I459, I49, I490, I491, I492, I493, I494, I495, I498, I499, I51, 1515, I516, I517, I518, I519, I52, I520, I521, I528, I64, I67, I678, I679, I68, I688, I69, I694, I698, I960, I988, I99

Neoplasms	GBD 4-digit codes:<br>C000, C001, C002, C003, C004, C005, C006, C008, C009, C020, C021, C022, C023, C024, C028, C029, C030, C031, C039, C040, C041, C048, C049, C050, C051, C052, C058, C059, C060, C061, C062, C068, C069, C080, C081, C088, C089, C090, C091, C098, C099, C100, C101, C102, C103, C104, C108, C109, C110, C111, C112, C113, C118, C119, C130, C131, C132, C138, C139, C150, C151, C152, C153, C154, C155, C158, C159, C160, C161, C162, C163, C164, C165, C166, C168, C169, C170, C171, C172, C173, C178, C179, C180, C181, C182, C183, C184, C185, C186, C187, C188, C189, C210, C211, C212, C218, C220, C221, C222, C223, C224, C227, C240, C241, C248, C249, C250, C251, C252, C253, C254, C257, C258, C259, C300, C301, C310, C311, C312, C313, C318, C319, C320, C321, C322, C323, C328, C329, C340, C341, C342, C343, C348, C349, C380, C381, C382, C383, C384, C388, C400, C401, C402, C403, C408, C409, C410, C411, C412, C413, C414, C418, C419, C430, C431, C432, C433, C434, C435, C436, C437, C438, C439, C440, C441, C442, C443, C444, C445, C446, C447, C448, C449, C450, C451, C452, C457, C459, C470, C471, C472, C473, C474, C475, C476, C478, C479, C480, C481, C482, C488, C490, C491, C492, C493, C494, C495, C496, C498, C499, C500, C501, C502, C503, C504, C505, C506, C508, C509, C510, C511, C512, C518, C519, C530, C531, C538, C539, C540, C541, C542, C543, C548, C549, C570, C571, C572, C573, C574, C578, C600, C601, C602, C608, C609, C620, C621, C629, C630, C631, C632, C637, C638, C670, C671, C672, C673, C674, C675, C676, C677, C678, C679, C680, C681, C688, C690, C691, C692, C693, C694, C695, C696, C698, C700, C701, C709, C710, C711, C712, C713, C714, C715, C716, C717, C718, C719, C720, C721, C722, C723, C724, C725, C728, C729, C750, C751, C752, C753, C754, C755, C758, C810, C811, C812, C813, C814, C817, C819, C820, C821, C822, C823, C824, C825, C826, C827, C829, C830, C831, C833, C835, C837, C838, C839, C840, C841, C844, C845, C846, C847, C848, C849, C851, C852, C857, C859, C860, C861, C862, C863, C864, C865, C866, C880, C882, C883, C884, C887, C889, C900, C901, C902, C903, C910, C912, C913, C916, C920, C921, C922, C923, C924, C925, C926, C930, C931, C933, C940, C942, C943, C944, C946, C947, C950, C951, C957, C959, C960, C962, C964, C965, C966, C967, C968, C969,<br>D000, D001, D002, D010, D011, D012, D013, D020, D021, D022, D023, D030, D031, D032, D033, D034, D035, D036, D037, D038, D039, D040, D041, D042, D043, D044, D045, D046, D047, D048, D049, D050, D051, D057, D059, D060, D061, D067, D069, D070, D071, D072, D074, D075, D090, D092, D093, D100, D101, D102, D103, D104, D105, D106, D107, D110, D117, D119, D120, D121, D122, D123, D124, D125, D126, D127, D128, D129, D130, D131, D132, D133, D134, D135, D136, D137, D140, D141, D142, D143, D150, D151, D152, D157, D159, D160, D161, D162, D163, D164, D165, D166, D167, D168, D169, D220, D221, D222, D223, D224, D225, D226, D227, D229, D230, D231, D232, D233, D234, D235, D236, D237, D239, D260, D261, D267, D269, D280, D281, D282, D287, D290, D291, D292, D293, D294, D297, D300, D301, D302, D303, D304, D307, D310, D311, D312, D313, D314, D315, D316, D319, D320, D321, D329, D330, D331, D332, D333, D334, D337, D339, D350, D351, D352, D353, D354, D355, D356, D357, D358, D359, D361, D367, D371, D372, D373, D374, D375, D380, D381, D382, D383, D384, D385, D391, D392, D400, D401, D407, D410, D411, D412, D413, D414, D417, D420, D421, D429, D430, D431, D432, D433, D434, D437, D439, D440, D441, D442, D443, D444, D445, D446, D447, D448, D460, D461, D462, D464, D465, D466, D467, D469, D470, D471, D472, D473, D474, D475, D477, D479, D480, D481, D482, D483, D484, D485, D486,<br>K620, K621, K635, N600, N601, N602, N603, N604, N608, N609, N840, N841, N870, N871, N872, N879

	GBD 3-digit codes:<br>C00, C01, C02, C03, CO4, C05, C06, C07, C08, C09, C10, C11, C12, C13, C15, C16, C17, C18, C19, C20, C21, C22, C23, C24, C25, C30, C31, C32, C33, C34, C37, C38, C40, C41, C43, C44, C45, C47, C48, C49, C50, C51, C52, C53, C54, C56, C57, C60, C61, C62, C63, C64, C65, C66, C67, C70, C71, C72, C73, C75, C81, C82, C83, C84, C85, C86, C88, C90, C91, C92, C93, C94, C95, C96, D03, D04, D05, D06, D10, D11, D12, D15, D16, D22, D23, D24, D26, D27, D31, D32, D33, D34, D35, D36, D42, D43, D45, D46, D47, D49, N60, N87
	Garbage codes redistributed:<br>C14, C140, C142, C148, C229, C26, C260, C261, C268, C269, C39, C390, C398, C399, C46, C460, C461, C462, C463, C467, C468, C469, C46, C55, C579, C639, C68, C689, C69, C699, C74, C740, C741, C749, C759, C760, C761, C762, C763, C764, C765, C767, C768, C770, C771, C772, C773, C774, C775, C778, C779, C780, C781, C782, C783, C784, C785, C786, C787, C788, C790, C791, C792, C793, C794, C795, C796, C797, C798, C799, C76, C77, C78, C79, C80, C800, C809, C911, C914, C915, C917, C918, C919, C927, C928, C929, C937, C939, C97, D00, D000, D01, D014, D015, D017, D019, D02, D024, D07, D073, D076, D09, D091, D097, D099, D10, D109, D13, D139, D14, D144, D17, D170, D171, D172, D173, D174, D175, D176, D177, D179, D18, D180, D181, D19, D190, D191, D197, D199, D20, D210, D211, D212, D213, D214, D215, D216, D219, D21, D28, D289, D29, D299, D30, D309, D36, D360, D369, D37, D370, D376, D377, D379, D38, D386, D39, D390, D397, D399, D40, D409, D41, D419, D44, D449, D48, D487, D489, E340

Garbage codes are ICD-10 codes considered too vague for meaningful public health use (e.g., unspecified cancer), or are classified as either imminent or immediate causes of death (e.g., septicemia), or unlikely to be an underlying cause of death (e.g., back pain) [1]. The GBD categorizes garbage codes into four levels based on the potential impact of misclassification on public health policy. Level 1 (very high implication) garbage codes could represent any of the three broad cause categories: communicable diseases, non-communicable diseases, or injuries. An example is septicemia. Level 2 (high implication) garbage codes likely correspond to one or two of the broad cause categories, such as essential (primary) hypertension. Level 3 (medium implication) garbage codes likely point to a cause within the same ICD chapter. For instance, unspecified cancer falls under ICD-10 Chapter II Neoplasms. Level 4 (low implication) garbage codes are likely associated with a single disease or injury category, such as unspecified stroke, which is categorized as a stroke death.

Appendix S2: ICD-10 codes of obesity-related conditions

Cause of death	ICD-10 codes
Chronic Kidney Disease	N18, N181, N182, N183, N184, N185, N189
Diabetes	E10, E100, E101, E102, E103, E104, E105, E106, E107, E108, E109, E11, E110, E111, E112, E113, E114, E115, E116, E117, E118, E119, E12, E120, E121, E122, E123, E124, E125, E126, E127, E128, E129, E13, E130, E131, E132, E133, E134, E135, E136, E137, E138, E139, E14, E140, E141, E142, E143, E144, E145, E146, E147, E148, E149
Hypertensive Heart Disease	I10, I11, I110, I119, I12, I120, I129, I13, I130, I131, I132, I139
Lipidemias	E78, E780, E781, E782, E783, E784, E785, E786, E788, E789
Obesity	E65, E66, E660, E661, E662, E668, E669

Appendix S3: Age-period-cohort modeling

Age-period-cohort modeling strategies can be defined as statistical attempts to partition variance into the unique components attributable to age, period, and cohort variations. Therefore, age, period, and cohort are often modeled as having a linear relationship with the outcome of interest, and each linear slope is estimated controlling for the additive contribution of the other two. These linear relationships are also termed "first-order effects." However, no statistical model can simultaneously estimate age, period, and cohort effects because of the collinearity among the three variables (Cohort = Period − Age). This collinearity results in a statistically non-identifiable design matrix, making simultaneous mathematical modeling of the linear functions of three effects impossible without additional restrictions in the model. We implemented the constraint provided by the intrinsic estimator to achieve identifiability.

The mortality rates were analyzed with a general age-period-cohort model specified as:

$$\log E(r_{ij}) = \log E\left(\frac{d_{ij}}{n_{ij}}\right) = \beta_0 + \beta_i^A + \beta_j^P + \beta_k^C$$

where $\log E(r_{ij})$ is the logarithm of the expected mortality rate based on d_{ij} deaths and n_{ij} person-years in cell ij of a cross-classification of deaths and person-years in age interval i (for $i = 1, \ldots, I$ age groups) and time period j (for $j = 1, \ldots, J$ periods). Age and period contributions are denoted by β_i^A and β_j^P, respectively. β_k^C denotes the kth (diagonal) birth cohort contribution (for $k = 1, \ldots, I + J$-1 birth cohorts), where the index $k = I - i + j$. For these data, $I = 10$ and $J = 5$ for $N = I \times J = 50$ age x period cells covering 14 birth cohorts. To provide an example of the tabular data used for the modeling, let's consider one cell corresponding to the 45-49 age group and the period group 1995-1999. The corresponding birth cohorts included in this cell are those in bold:

Age / Period	1995	1996	1997	1998	1999
45	**1950**	**1951**	**1952**	**1953**	**1954**
46	**1949**	**1950**	**1951**	**1952**	**1953**
47	**1948**	**1949**	**1950**	**1951**	**1952**
48	**1947**	**1948**	**1949**	**1950**	**1951**
49	**1946**	**1947**	**1948**	**1949**	**1950**

The birth cohort groupings are the diagonals of a super-matrix built similarly as in this example but spanning all age groups and period groups.

To estimate the model above, the intrinsic estimator imposes an ANOVA type centered-coefficients normalization $\sum \beta_i^A = \sum \beta_j^P = \sum \beta_k^C = 0$.

Under this normalization, the estimates reflect the age, birth cohort, and time period deviations from the grand mean mortality rate for each population, net of the other age-period-cohort contributions. The formal definitions of the intrinsic estimator and its properties as a statistical estimator are described in detail elsewhere [2]. Under the ANOVA type constraints, an omitted cell must still be chosen to identify the model. We fit one age-period-cohort model using the intrinsic estimator constraint that omits the last age-period-cohort cell in main analyses.

Appendix S4: Sensitivity analysis with re-allocation of garbage codes

We re-distributed some garbage codes – those falling into the levels 1 and 2 – listed as the UCOD to CVD. We focused on CVD rather than cancer as the portion of overall garbage codes potentially related to CVD is higher than that for cancer [3,4]. Following a previous study [5], we considered essential hypertension (I10), cardiac arrest (I46), heart failure (I50), atherosclerosis (I70) and deaths coded with R00-R94, R96-R99 and applied a redistribution algorithm based on the probability of CVD as the true underlying cause of death. The estimated probabilities were drawn from the GBD 2010 study [3]. We re-calculated the obesity-related and obesity-unrelated age-standardized CVD mortality rates after redistribution of these codes.

Between 1995 and 2019, there were 860 893 (49.8%) deaths related to CVD either as the underlying or as a concomitant cause of death, a considerably higher portion than when the above garbage codes were not considered (40.5%). This indicates that the considered garbage codes were frequent and supports their choice for the sensitivity analysis.

Appendix S5: Sensitivity analysis with a different specification of the intrinsic estimator for the age-period-cohort model

We assessed the validity of the period-based and cohort-based trends estimated from the intrinsic estimator of age-period-cohort models by changing the referent categories as suggested in Masters et al., 2016 [6]. Specifically, age-period-cohort models were fitted with the intrinsic estimator using last age-period-cohort categories as referents, while first categories were used in main analyses.

Appendix S6: Sensitivity analysis with a different definition of obesity-related cancers

We selected 12 cancer types on the basis of their association with obesity according to the International Agency for Cancer Research. This viewpoint was implemented in previous studies examining trends of obesity-related cancer mortality and incidence in the United States [7,8]. Specifically, the cancers were: colorectal, oesophageal (adenocarcinoma), gallbladder, gastric cardia, kidney, liver and intrahepatic bile duct, multiple myeloma, pancreatic, meningioma and thyroid cancer, and, in women, uterine corpus (including endometrial cancer and uterine sarcoma), breast, and ovarian cancer. We considered the ICD-10 codes used in Avery et al [7].

We estimated the prevalence of these codes among the MCOD-based obesity-related and -unrelated cancer deaths, identified as described in the main text. For simplicity, we examined only the code reported for the UCOD of these deaths. To test for the differential prevalence, we ran a Fisher test in R software, using the function "fisher.test".

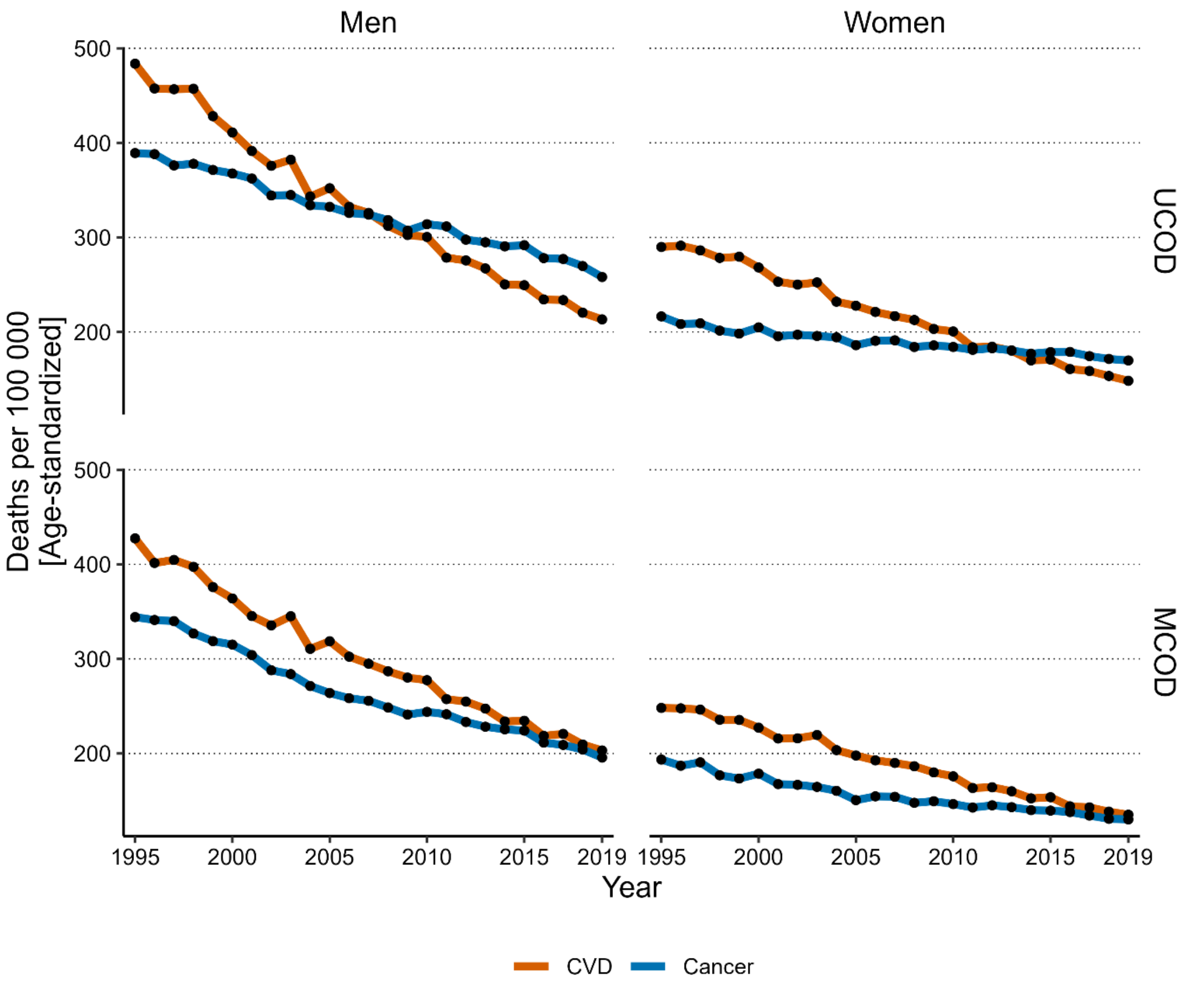

Figure S1: CVD/cancer deaths by sex and by weighting method of death causes. While the traditional approach assigns a weight of one to the underlying cause of death (UCOD) and zero to all other causes present in the certificate, the weighted MCOD approach distributes fractional weights across all causes and maintains the individual death as the unit of analysis with weights summing to one. As reported in the main text, the selected MCOD approach in main analysis was the so-called double UCOD.

There were 472 561 / 640 617 deaths related to UCOD / MCOD CVD and 448 592 / 459 222 to UCOD / MCOD cancer.

Annual percentage changes for UCOD CVD: -3.4% (-3.5% to -3.2%) between 1995 and 2019 among men; -2.1% (95% CI: -2.8% to -1.6%) between 1995 and 2001, -3.1% (95% CI: -3.5% to -3.0%) between 2001 and 2019 among women.

Annual percentage changes for UCOD Cancer: -1.6% (-1.7% to -1.5%) between 1995 and 2019 among men; -0.85% (95% CI: -0.95% to -0.75%) between 1995 and 2019 among women.

Compared to MCOD-based annual percentage change (reported in the main analysis), UCOD CVD changes were similar, while they were smaller for UCOD cancer and no attenuation of decline was observed.

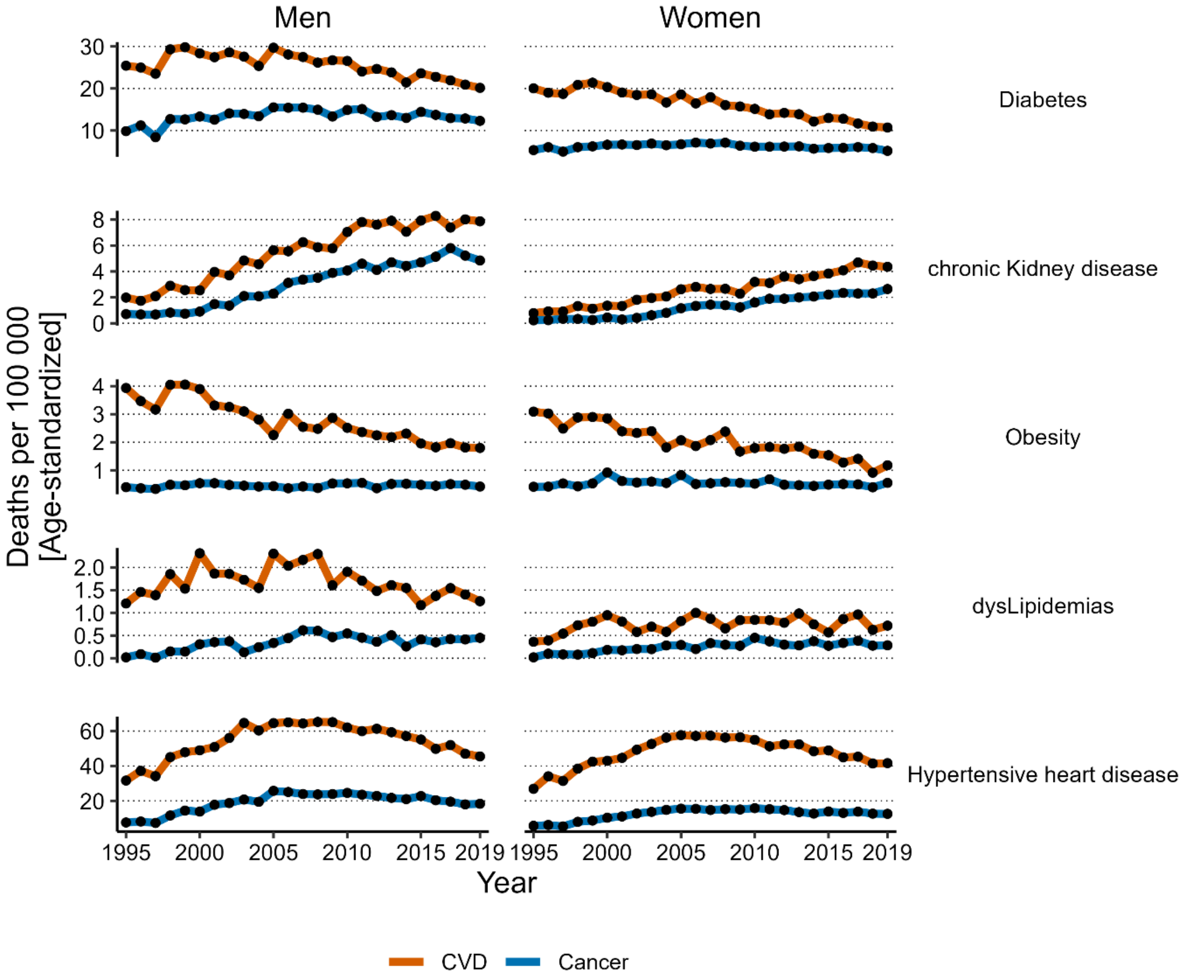

Figure S2: CVD/cancer mortality rates by individual DKOLH condition. Mortality rates are age-standardized.

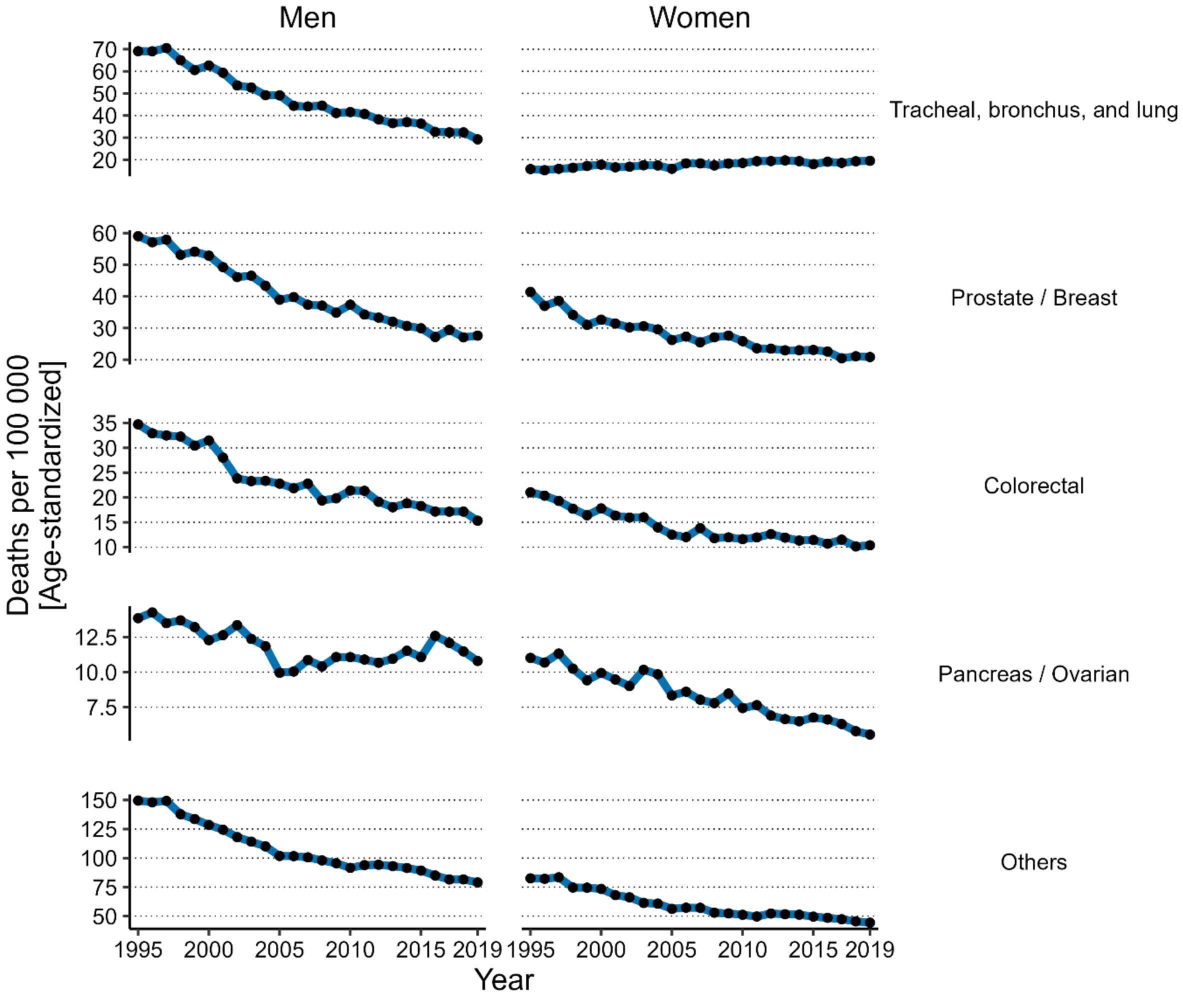

Figure S3: Non-DKOLH cancer mortality rates by specific cancer sites. Mortality rates are age-standardized. The sex-specific top four types of cancer deaths in Switzerland were chosen. Among men: i) tracheal, bronchus, and lung; ii) prostate; iii) colorectal; iv) pancreatic. Among women: i) tracheal, bronchus, and lung; ii) breast; iii) colorectal; iv) ovarian. The category Others includes all other cancer sites.

For tracheal, bronchus, and lung cancer, the annual percentage change was -3.5% (95% CI: -3.8% to -3.3%) among men and 0.9% (95% CI: 0.6% to 1.2%) among women.
For prostate cancer, the annual percentage change was -3.4% (95% CI: -3.6% to -3.2%).
For breast cancer, the annual percentage change was -4.3% (95% CI: -5.4% to -2.7%) between 1995 and 2001, while -2.2% (95% CI: -2.6% to -1.1%) between 2001 and 2019.
For colorectal cancer, the annual percentage change was -4.7% (95% CI: -6.5% to -3.4%) between 1995 and 2004, while -2.4% (95% CI: -3.0% to -0.7%) between 2004 and 2019 among men; and -4.4% (95% CI: -6.5% to -3.4%) between 1995 and 2006, while -1.5% (95% CI: -2.3% to 0.4%) between 2006 and 2019 among women.
For pancreatic cancer, the annual percentage change was -2.8% (95% CI: -4.4% to -1.9%) between 1995 and 2006, while 0.7% (95% CI: -0.1% to 1.8%) between 2006 and 2019.
For ovarian cancer, the annual percentage change was -2.7% (95% CI: -3.0% to -2.4%).
For other cancers, the annual percentage change was -3.9% (95% CI: -4.7% to -3.4%) between 1995 and 2005, while -1.8% (95% CI: -2.2% to -1.4%) between 2005 and 2019 among men; and -4.0% (95% CI: -5.3% to -3.2%) between 1995 and 2005, while -1.7% (95% CI: -2.1% to -0.8%) between 2005 and 2019 among women.

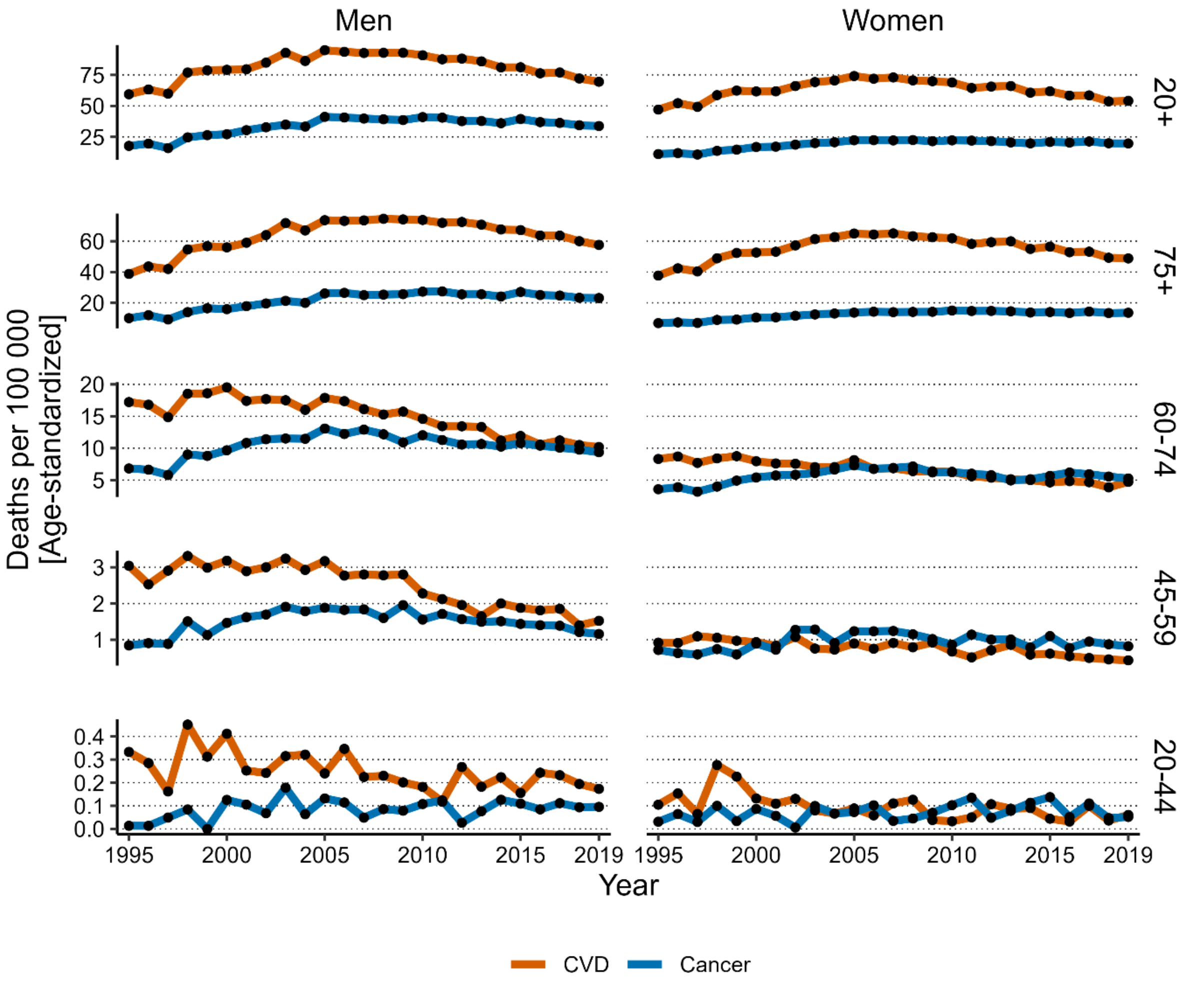

Figure S4: DKOLH CVD/cancer mortality rates by sex and age group. Mortality rates are age-standardized.

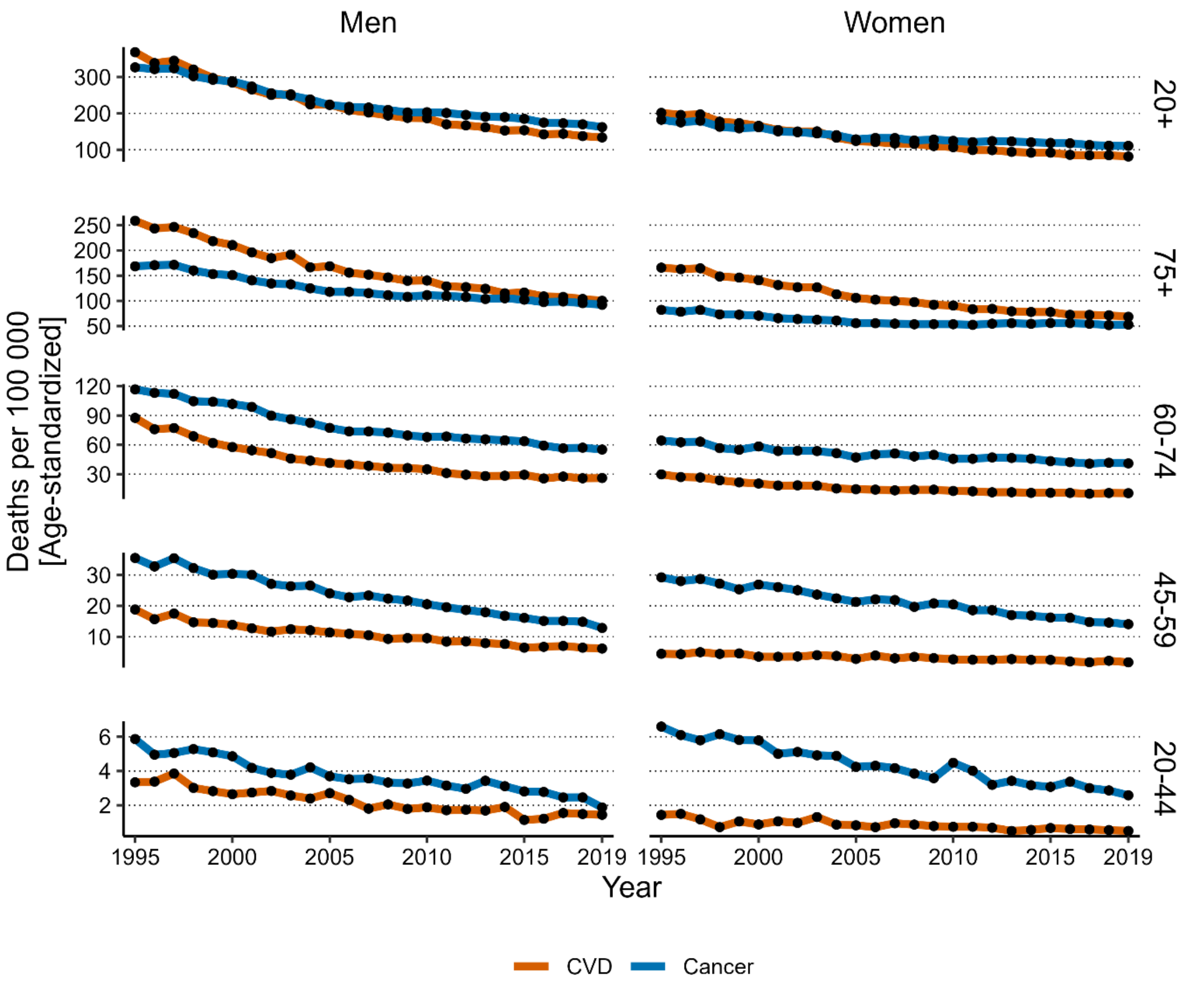

Figure S5: Non-DKOLH CVD/cancer mortality rates by sex and age group. Mortality rates are age-standardized.

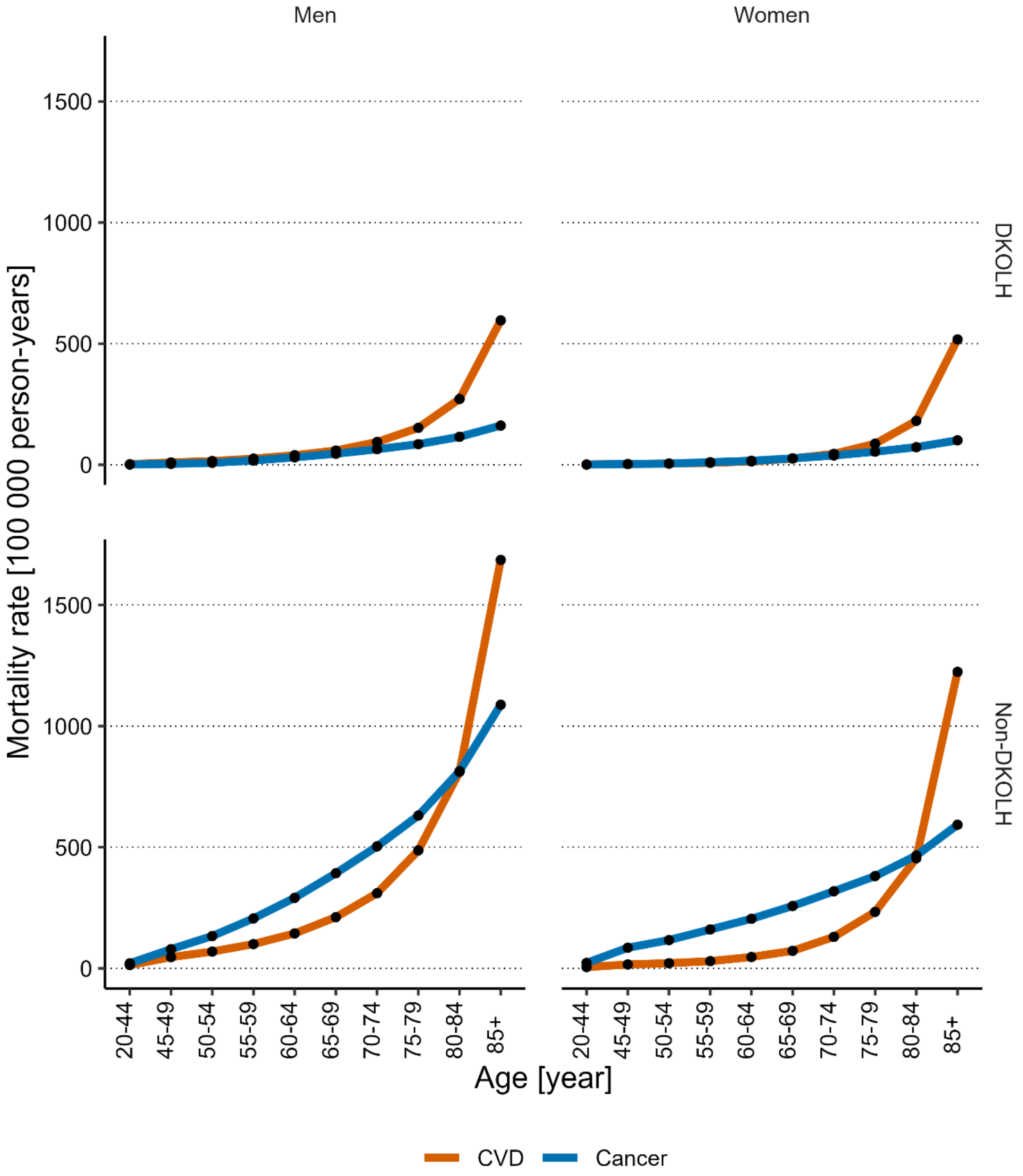

Figure S6: Age-based variations in CVD and cancer mortality rates by sex and obesity.

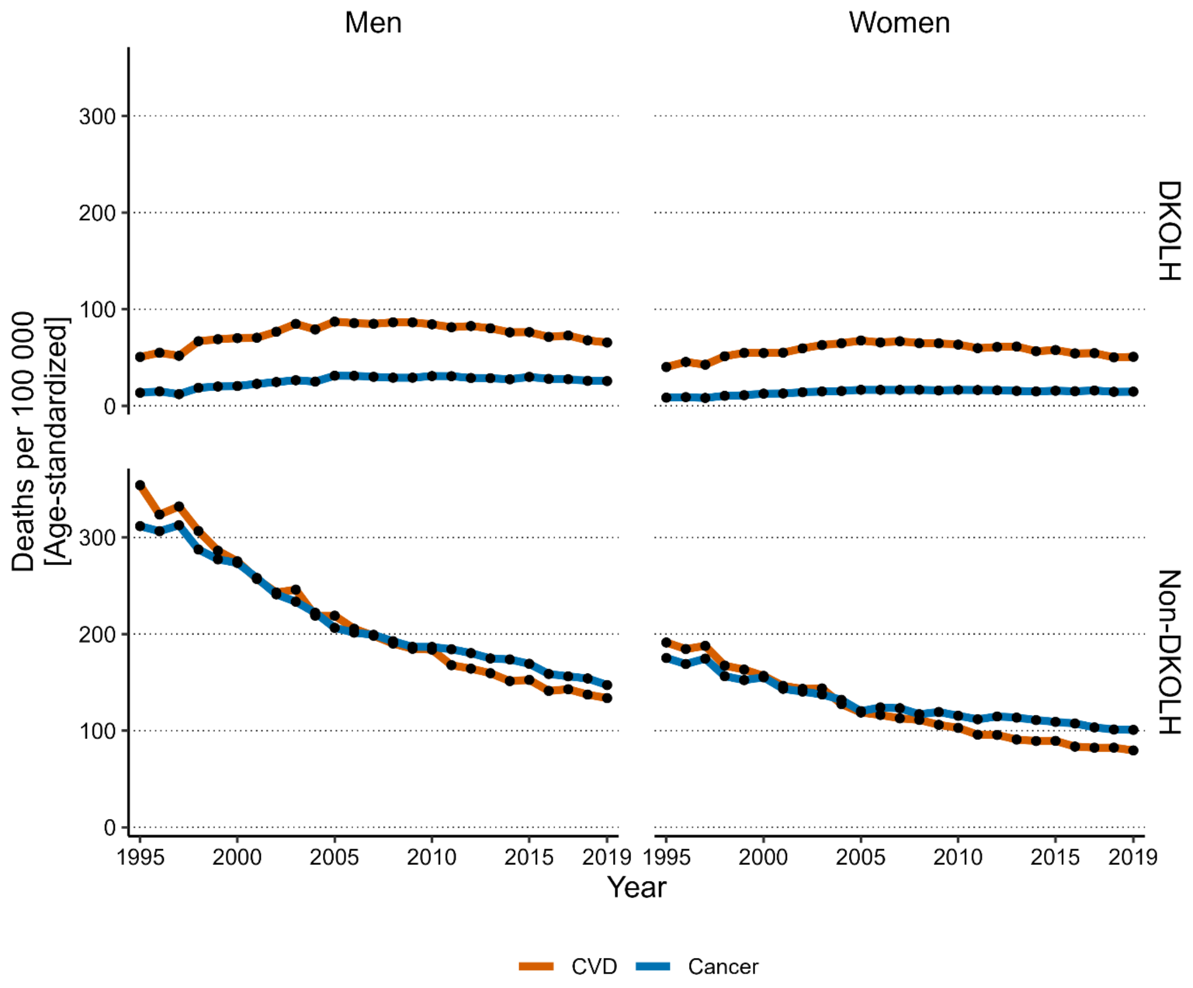

Figure S7: DKOLH (top) and non-DKOLH (bottom) CVD and cancer mortality rates from 1995-2019 by sex (men on the left, women on the right). Mortality rates are age-standardized and computed by applying an equal weight across the multiple causes of death.

Annual percentage changes for DKOLH CVD: 5.8% (4.7% to 7.1%) between 1995 and 2005, -1.9% (95% CI: -2.7% to -1.3%) between 2005 and 2019 among men; 5.2% (95% CI: 4.3% to 6.2%) between 1995 and 2005, -2.1% (95% CI: -2.6% to -1.6%) between 2005 and 2019 among women.

Annual percentage changes for non-DKOLH CVD: -4.8% (-5.7% to -4.4%) between 1995 and 2006, -3.3% (95% CI: -3.7% to -2.6%) between 2006 and 2019 among men; -4.3% (95% CI: -4.8% to -4.0%) between 1995 and 2011, -2.3% (95% CI: -3.1% to -1.4%) between 2011 and 2019 among women.

Annual percentage changes for DKOLH cancer: 9% (7.1% to 11.5%) between 1995 and 2005, -1.1% (95% CI: -2.3% to 0%) between 2005 and 2019 among men; 7.6% (95% CI: 6.4% to 9%) between 1995 and 2005, -1% (95% CI: -1.7% to -0.3%) between 2005 and 2019 among women.

Annual percentage changes for non-DKOLH cancer: -4.2% (-4.9% to -3.7%) between 1995 and 2006, -2.3% (95% CI: -2.7% to -1.7%) between 2006 and 2019 among men; -3.4% (95% CI: -4.3% to -2.9%) between 1995 and 2005, -1.5% (95% CI: -1.8% to -1.1%) between 2005 and 2019 among women.

Among men, the proportion of DKOLH rates to the overall CVD mortality rate was 12.5% in 1995 and 32.9% in 2019. Among women, the proportion rose from 17.3% in 1995 to 38.9% in 2019.

Among men, the proportion of DKOLH rates to the overall cancer mortality rate was 4.2% in 1995 and 14.8% in 2019. Among women, the proportion rose from 4.6% in 1995 to 12.7% in 2019.

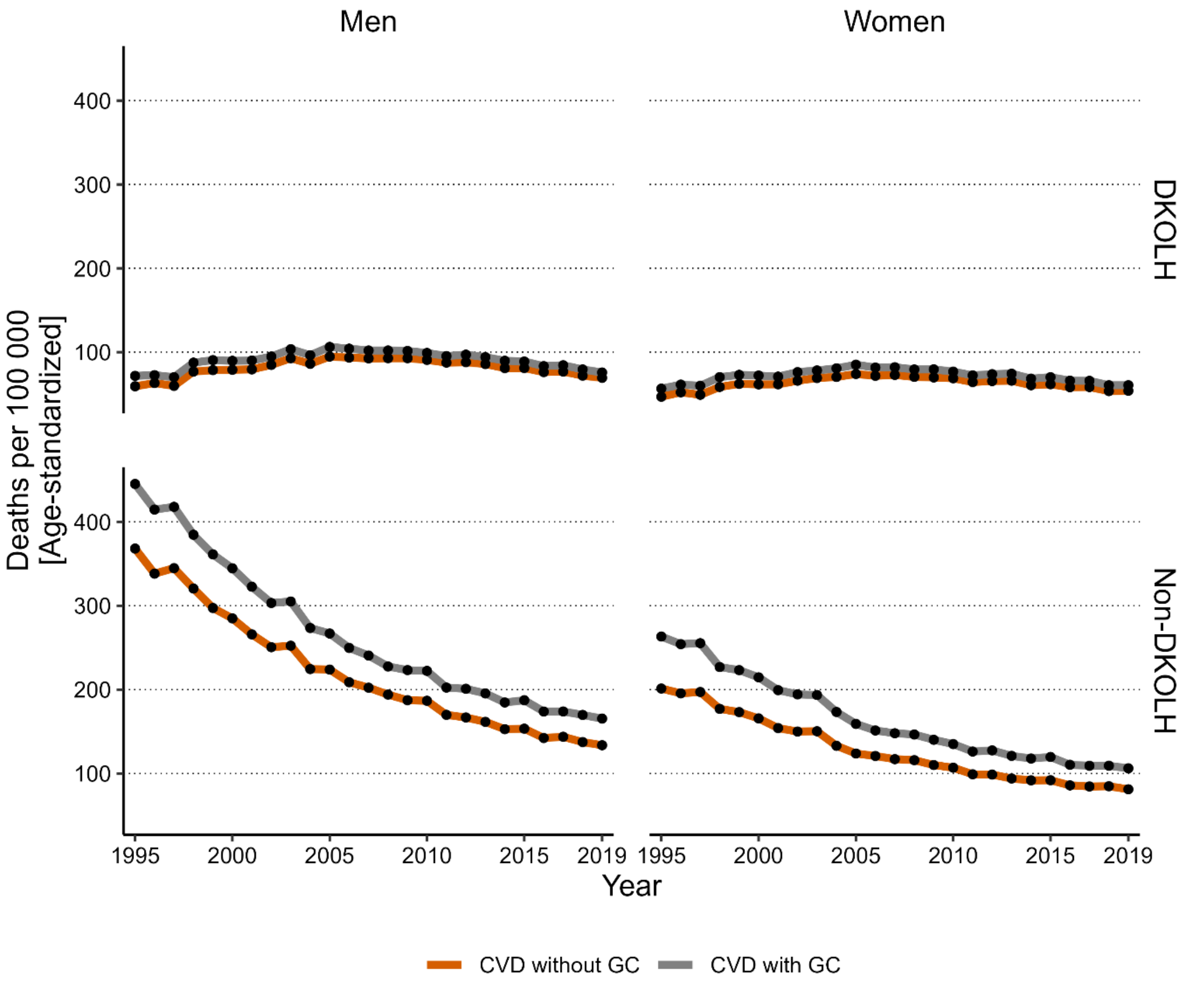

Figure S8: DKOLH (top) and non-DKOLH (bottom) CVD mortality rates without reassigned garbage codes (GC, in orange) and with reassigned garbage codes (in gray) from 1995-2019 by sex (men on the left, women on the right). Mortality rates are age-standardized.

Annual percentage changes for DKOLH CVD with GC: 4.3% (3.3% to 5.4%) between 1995 and 2005, -2.2% (95% CI: -2.8% to -1.6%) between 2005 and 2019 among men; 3.8% (95% CI: 3.0% to 4.8%) between 1995 and 2005, -2.3% (95% CI: -2.8% to -1.8%) between 2005 and 2019 among women.

Annual percentage changes for non-DKOLH CVD with GC: -5.1% (-5.5% to -4.8%) between 1995 and 2008, -3.0% (95% CI: -3.4% to -2.4%) between 2008 and 2019 among men; -4.8% (95% CI: -5.6% to -4.3%) between 1995 and 2009, -2.6% (95% CI: -3.3% to -1.4%) between 2009 and 2019 among women.

When rates were estimated considering GC, among men, the proportion of obesity-related rates to the overall CVD mortality rate was 13.9% in 1995 and 31.4% in 2019. Among women, the proportion rose from 17.7% in 1995 to 36.4% in 2019.

Finally, we estimated the annual percentage changes as well for overall CVD mortality when rates were estimated considering GC. Among men, the rates declined steadily at a pace of 3.1% (3.0% to 3.3%). Among women, the rates declined at a pace of 2.8% (2.7% to 2.9%).

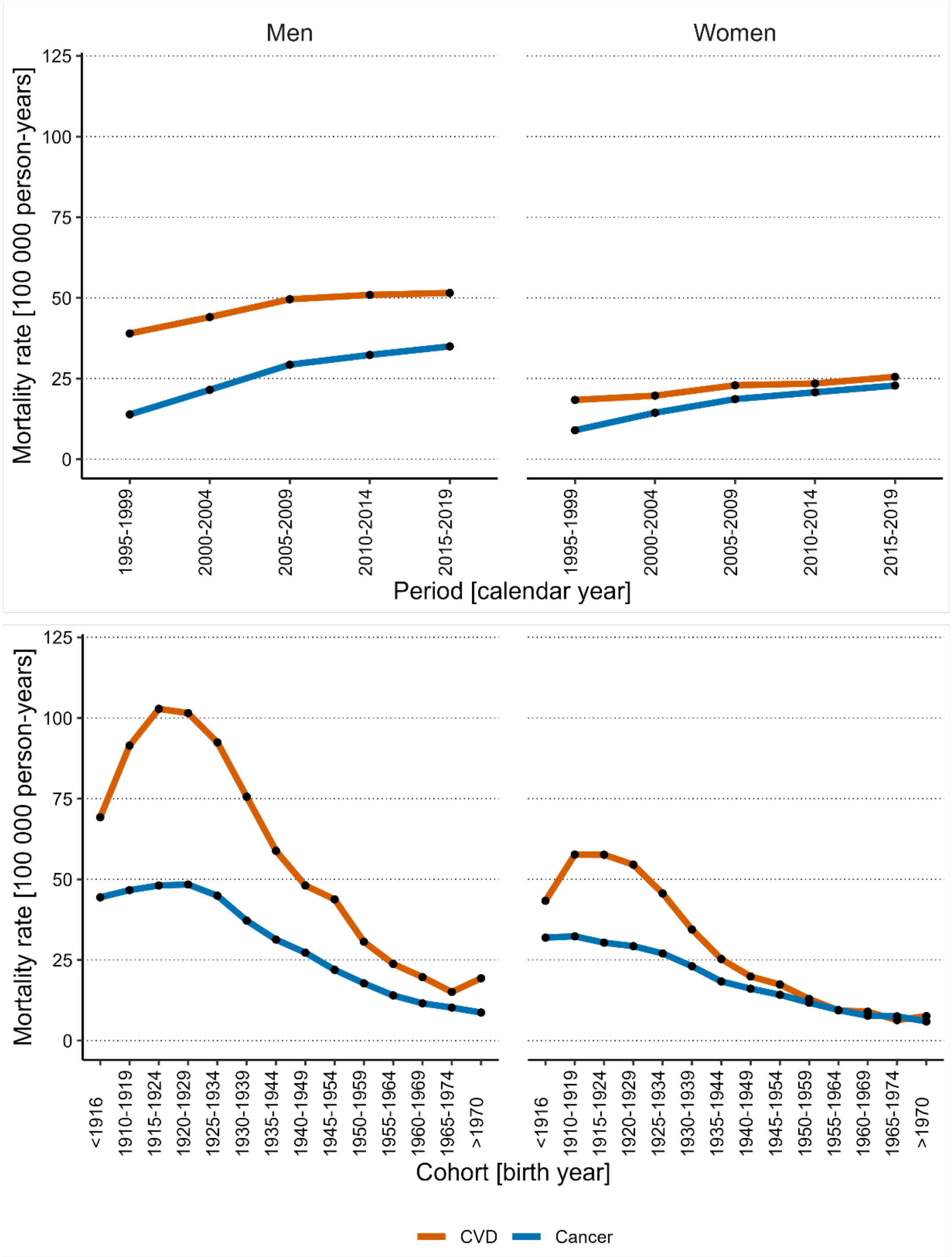

Figure S9: Period-based (top) and cohort-based (bottom) variations in DKOLH CVD and cancer mortality rates by sex (men on the left, women on the right). APC models were fitted with the intrinsic estimator using last age-period-cohort categories as referents (first categories were used in main analyses).

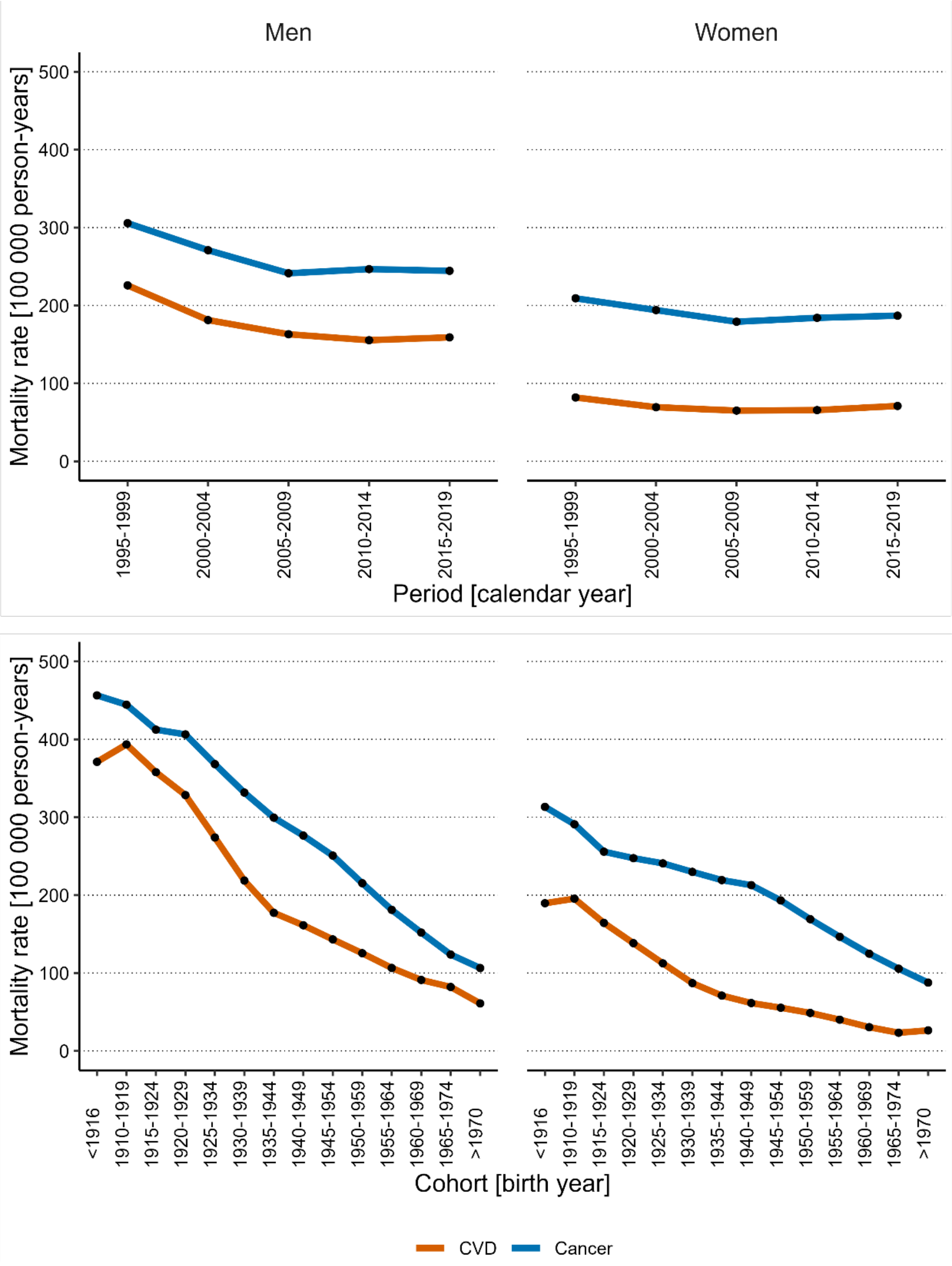

Figure S10: Period-based (top) and cohort-based (bottom) variations in non-DKOLH CVD and cancer mortality rates by sex (men on the left, women on the right). APC models were fitted with the intrinsic estimator using last age-period-cohort categories as referents (first categories were used in main analyses).

References

1. Iburg KM, Mikkelsen L, Adair T, Lopez AD. Are cause of death data fit for purpose? evidence from 20 countries at different levels of socio-economic development. *PLoS One*. 2020;15(8):e0237539. doi:10.1371/journal.pone.0237539
2. Fosse E, Winship C. Moore–Penrose Estimators of Age–Period–Cohort Effects: Their Interrelationship and Properties. *Sociological Science*. 2018;5(14):304-334. doi:10.15195/v5.a14
3. Lozano R, Naghavi M, Foreman K, et al. Global and regional mortality from 235 causes of death for 20 age groups in 1990 and 2010: a systematic analysis for the Global Burden of Disease Study 2010. *The Lancet*. 2012;380(9859):2095-2128. doi:10.1016/S0140-6736(12)61728-0
4. Anker D, Cullati S, Rod NH, Chiolero A, Carmeli C. Intergenerational educational trajectories and premature mortality from chronic diseases: A registry population-based study. *SSM Popul Health*. Dec 2022;20:101282. doi:10.1016/j.ssmph.2022.101282
5. Stolpe S, Kowall B, Stang A. Decline of coronary heart disease mortality is strongly effected by changing patterns of underlying causes of death: an analysis of mortality data from 27 countries of the WHO European region 2000 and 2013. *Eur J Epidemiol*. Jan 2021;36(1):57-68. doi:10.1007/s10654-020-00699-0
6. Masters RK, Powers DA, Hummer RA, Beck A, Lin S-F, Finch BK. Fitting Age-Period-Cohort Models Using the Intrinsic Estimator: Assumptions and Misapplications. *Demography*. 2016;53(4):1253-1259.
7. Avery CL, Howard AG, Nichols HB. Comparison of 20-Year Obesity-Associated Cancer Mortality Trends With Heart Disease Mortality Trends in the US. *JAMA Netw Open*. May 3 2021;4(5):e218356. doi:10.1001/jamanetworkopen.2021.8356
8. Sung H, Siegel RL, Rosenberg PS, Jemal A. Emerging cancer trends among young adults in the USA: analysis of a population-based cancer registry. *The Lancet Public Health*. 2019;4(3):e137-e147. doi:10.1016/S2468-2667(18)30267-6
